# Three new *Curvularia* species from clinical and environmental sources

**DOI:** 10.3897/mycokeys.68.51667

**Published:** 2020-06-17

**Authors:** Isabel Iturrieta-González, Josepa Gené, Nathan Wiederhold, Dania García

**Affiliations:** 1 Unitat de Micologia, Facultat de Medicina i Ciències de la Salut and IISPV, Universitat Rovira i Virgili, Reus, Spain; 2 Fungus Testing Laboratory, Department of Pathology and Laboratory Medicine, University of Texas Health Science Center, San Antonio, TX, USA

**Keywords:** *
Ascomycetes
*, Dematiaceous hyphomycetes, phylogeny, Pleosporaceae, taxonomy

## Abstract

*Curvularia* is a Pleosporalean monophyletic genus with a great diversity of species, including relevant phytopathogenic, animal and human pathogenic fungi. However, their microscopic identification is difficult due to overlapping morphological features amongst species. In recent years, multi-locus sequence analysis using the ITS region of the rDNA and fragments of the genes *gapdh* and *tef*1 revealed numerous cryptic species, especially in isolates that commonly produced 3-septate conidia. Therefore, based on sequence analysis of the above-mentioned DNA barcodes recommended for species delineation in *Curvularia*, we propose three novel species, *C. paraverruculosa*, *C. suttoniae* and *C. vietnamensis*, isolated from soil, human clinical specimens and plant material, respectively, collected in different countries. These new species are morphologically characterised and illustrated in the present study. *Curvularia paraverruculosa* differs from its counterparts, *C. americana* and *C. verruculosa*, mainly by its narrower conidia. *Curvularia suttoniae* and *C. vietnamensis* are closely related to *C. petersonii*, but the former two have larger conidia.

## Introduction

The genus *Curvularia*Boedijn (1933), typified by *C. lunata* (Wakker) Boedijn, belongs in Pleosporaceae, Pleosporales (Wijayawardene et al. 2018). Members of *Curvularia* show different life modes, i.e. saprophytic, endophytic and also pathogenic on plants and animals (Marin-Felix et al. 2017a). Phytopathogenic species can affect wild grasses and staple crops, such as rice, maize, wheat or sorghum and give rise to serious losses in agricultural production (Gautam et al. 2013, Manamgoda et al. 2015, Marin-Felix et al. 2017a, Tan et al. 2018). The endophytic species have garnered interest in recent years for their use in the production of bio-based products that are beneficial to living organisms and the environment (Bengyella et al. 2019). Since the first report of *Curvularia* as a human pathogen in a patient with mycetoma (Baylet et al. 1959), other clinical presentations have been reported, such as superficial and deep infections that mainly affect the respiratory tract but can even cause cerebral phaeohyphomycosis with an extremely poor prognosis (de Hoog et al. 2000).

The genus is morphologically distinguished mainly by its asexual morph, which shows sympodial conidiophores with mono- to polytretic conidiogenous cells and transversally septate conidia. Typically, the conidia in *Curvularia* are curved due to the hypertrophy of one of the intermediate cells and they are euseptate (Ellis 1971), although other authors opine that the conidia in *Curvularia* are distoseptate (Sivanesan 1987, Seifert et al. 2011, Madrid et al. 2014). The species of *Bipolaris* and *Exserohilum* have typically straight and distoseptate conidia; however, some of them have been transferred to *Curvularia*, based on their DNA sequence analyses (Manamgoda et al. 2012, Hernández-Restrepo et al. 2018, Tan et al. 2018). Furthermore, due to the overlapping of morphological characters amongst certain species of *Curvularia*, such as conidial size, shape and septation, an accurate identification at the species level is difficult without a DNA sequence analysis (da Cunha et al. 2013, Madrid et al. 2014, Manamgoda et al. 2015). Several cryptic species have been described recently using only multi-locus sequence analyses of the recommended DNA barcodes for species delimitation, i.e. the internal transcribed spacer (ITS) region of the rDNA and the protein-coding loci glyceraldehyde-3-phosphate dehydrogenase (*gapdh*) and translation elongation factor 1-a (*tef*1) (Marin-Felix et al. 2017a, Tan et al. 2018). Nearly 130 species have so far been accepted in *Curvularia*, including the species classified previously in the teleomorphic genera *Cochliobolus* and *Pseudocochliobolus* after applying the current criteria for fungal nomenclature (Manamgoda et al. 2012, 2015, Madrid et al. 2014, Hyde et al. 2017, Marin-Felix et al. 2017a, 2017b, Dehdari et al. 2018, Heidari et al. 2018, Hernández-Restrepo et al. 2018, Liang et al. 2018, Mehrabi-Koushki et al. 2018, Tan et al. 2018, Tibpromma et al. 2018, Kiss et al. 2019, Raza et al. 2019, Zhang et al. 2020).

Based on a polyphasic approach, combining morphological and phylogenetic analyses, three novel *Curvularia* species are proposed here, isolated from human clinical specimens in the USA, soil in Mexico and seed and plant debris in Vietnam and Indonesia, respectively.

## Material and methods

### Origin of isolates

Five unidentified *Curvularia* isolates, maintained in the fungal collection of the Medical School of the Rovira i Virgili University (FMR; Reus, Spain), were included in the study. Two of these (FMR 10992, FMR 11690) were isolated from human specimens in the USA by Deana A. Sutton of the Fungus Testing Laboratory at the University of Texas Health Sciences Center (UTHSC; San Antonio, USA) and the other three (FMR 11956, FMR 17656, FMR 17659) were isolated from environmental samples; the first from sorghum seeds collected in Indonesia, the second from soil collected in the Mexican region of Michoacán and the third from unidentified plant material collected in the north-east of Vietnam.

### DNA extraction, PCR, sequencing and phylogenetic analysis

The fungal DNA was extracted from colonies growing on potato dextrose agar (PDA; Pronadisa, Madrid, Spain) for 7 to 10 days at 25 °C in darkness and following the protocol of Müller et al. (1998). The ITS barcode, including the 5.8S gene and the genes *gapdh* and *tef*1 were analysed following Marin-Felix et al. (2017a). Amplification was carried out using the primer pairs ITS5/ITS4 for the ITS region (White et al. 1990), gpd1/gpd2 for *gapdh* (Berbee et al. 1999) and EF983/2218R for *tef*1 (Schoch et al. 2009). The PCR products were purified and stored at -20 °C until sequencing. The same pairs of primers used for the amplification were also used to obtain the DNA sequences, which were processed at Macrogen Europe (Macrogen Inc., Madrid, Spain). The sequences of each isolate were edited using SeqMan v. 7.0.0 (DNAStar Lasergene, Madison, WI, USA) to obtain the consensus sequences.

We made a preliminary comparison of *gapdh* sequences generated from our isolates with those of the National Center for Biotechnology Information (NCBI) using the Basic Local Alignment Search Tool (BLASTn) for their molecular identification. To establish the phylogenetic position of unidentified isolates with respect to the most accepted species in *Curvularia*, we carried out individual (data not shown) and combined alignments of the three loci complemented by all available sequences of the ex-type and reference strains of *Curvularia* species retrieved from NCBI (Table 1). Based on this first phylogeny of the genus, a more restricted multi-locus analysis was carried out, including only those *Curvularia* species most related to the isolates under study. The alignments were made in the MEGA (Molecular Evolutionary Genetics Analysis) software v.6.0. (Tamura et al. 2013), using ClustalW algorithm (Thompson et al. 1994), refined with MUSCLE (Edgar 2004) in the same platform and manually adjusted as necessary. Phylogenetic reconstructions were made using Maximum Likelihood (ML) and Bayesian Inference (BI) approaches under RAxML-HPC2 on XSEDE v.8.2.12 (Stamatakis et al. 2014) in CIPRES Science gateway portal (Miller et al. 2010) and MrBayes v. 3.2.6 (Ronquist et al. 2012), respectively.

**Table 1.** Species included in this study, their substrate, origin and GenBank accession numbers.

Species	Strain no^1^	Substrate	Country	Genbank accession no.^2^		
				ITS	*gapdh*	*tef*1
*Bipolaris maydis*	CBS 136.29 T	*Zea mays*	USA	AF071325	KM034846	KM093794
*B. saccharicola*	CBS 155.26 T	Unknown	Unknown	KY905674	KY905686	KY905694
*Curvularia aeria*	CBS 294.61 T	Air	Brazil	HF934910	HG779148	–
*C. affinis*	CBS 154.34 T	Unknown	Indonesia	KJ909780	KM230401	KM196566
*C. ahvazensis*	CBS 144673 T	*Zinnia elegans*	Iran	KX139029	MG428693	MG428686
*C. akaii*	CBS 317.86	Themada triandra subsp. japonica	Japan	KJ909782	KM230402	KM196569
*C. akaiiensis*	BRIP 16080 T	Unknown	India	KJ415539	KJ415407	KJ415453
*C. alcornii*	MFLUCC 10-0703 T	*Z. mays*	Thailand	JX256420	JX276433	JX266589
*C. americana*	UTHSC 08-3414 T	Human ankle	USA	HE861833	HF565488	–
	UTHSC 07-2649	Human toe tissue	USA	HE861834	HF565486	–
	UTHSC 08-84	Human nasal sinus	USA	HG779015	HG779115	–
	UTHSC 08-278	Human peritoneal dialysis fluid	USA	HE861832	HF565487	–
	UTHSC 08-2697	Human leg	USA	HG779016	HG779117	–
*C. annelliconidiophori*	CGMCC3.19352 T	Roots of *Saccharum officinarum*	China	MN215641	MN264077	MN263935
*C. asiatica*	MFLUCC 10-0711 T	*Panicum* sp.	Thailand	JX256424	JX276436	JX266593
*C. australiensis*	BRIP 12044 T	*Oryza sativa*	Australia	KJ415540	KJ415406	KJ415452
	CBS 172.57	*O. sativa* seeds	Vietnam	JN601026	JN601036	JN601003
*C. australis*	BRIP 12521 T	*Sporobolus caroli*	Australia	KJ415541	KJ415405	KJ415451
*C. bannonii*	BRIP 16732 T	*Jacquemontia tamnifolia*	USA	KJ415542	KJ415404	KJ415450
*C. beasleyi*	BRIP 10972 T	*Chloris gayana*	Australia	MH414892	MH433638	MH433654
	BRIP 15854	*Leersia hexandra*	Australia	MH414893	MH433639	MH433655
*C. beerburrumensis*	BRIP 12942 T	*Eragrostis bahiensis*	Australia	MH414895	MH433634	MH433657
*C. boeremae*	IMI 164633 T	*Portulaca oleracea*	India	MH414911	MH433641	–
*C. borreriae*	CBS 859.73	Volcanic ash soil	Chile	HE861848	HF565455	–
*C. bothriochloae*	BRIP 12522 T	*Bothriochloa bladhii*	Australia	KJ415543	KJ415403	KJ415449
*C. brachyspora*	CBS 186.50	Soil	Indonesia	KJ922372	KM061784	KM230405
*C. buchloes*	CBS 246.49 T	*Buchloë dactyloides*	USA	KJ909765	KM061789	KM196588
*C. carica-papayae*	CBS 135941 T	*Carica papaya*	India	HG778984	HG779146	–
*C. chiangmaiensis*	CPC 28829 T	*Z. mays*	Thailand	MF490814	MF490836	MF490857
*C. chlamydospora*	UTHSC 07-2764 T	Human toe nail	USA	HG779021	HG779151	–
*C. chonburiensis*	MFLUCC 16-0375 T	Dead leaf of *Pandanus* sp.	Thailand	MH275055	MH412747	–
*C. clavata*	BRIP 61680	*Oryza* sp.	Australia	KU552205	KU552167	KU552159
*C. cymbopogonis*	CBS 419.78	*Yucca* leaf spot	Netherlands	HG778985	HG779129	–
*C. coatesiae*	BRIP 24261 T	*Litchi chinensis*	Australia	MH414897	MH433636	MH433659
*C. coicis*	CBS 192.29 T	*Coix lacryma-jobi*	Japan	AF081447	AF081410	JN601006
*C. coimbatorensis*	SZMC 22225 T	Human corneal scraping	India	MN628310	MN628306	MN628302
*C. colbranii*	BRIP 13066 T	*Crinum zeylanicum*	Australia	MH414898	MH433642	MH433660
*C. comoriensis*	CBS 110673	Unknown	Unknown	LT631357	LT715841	–
*C. crassiseptum*	CBS 503.90 T	Plant material	Nigeria	LT631310	LT715882	–
*C. crustacea*	BRIP 13524 T	*Sporobolus* sp.	Indonesia	KJ415544	KJ415402	KJ415448
*C. dactyloctenicola*	CPC 28810 T	*Dactyloctenium aegyptium*	Thailand	MF490815	MF490837	MF490858
*C. dactyloctenii*	BRIP 12846 T	*Dactyloctenium radulans*	Australia	KJ415545	KJ415401	KJ415447
*C. deightonii*	CBS 537.70	*Sorghum vulgare*	Denmark	LT631356	LT715839	–
*C. determinata*	CGMCC3.19340 T	Leaves of *S. officinarum*	China	MN215653	MN264088	MN263947
*C. elliptiformis*	CGMCC3.19351 T	Roots of *S. officinarum*	China	MN215656	MN264091	MN263950
*C. ellisii*	CBS 193.62 T	Air	Pakistan	JN192375	JN600963	JN601007
*C. eragrosticola*	BRIP 12538 T	*Eragrostis pilosa*	Australia	MH414899	MH433643	MH433661
*C. eragrostidis*	CBS 189.48	*Sorghum* seed	Indonesia	HG778986	HG779154	–
*C. falsilunata*	CGMCC3.19329 T	Roots of *S. officinarum*	China	MN215660	MN264093	MN263954
*C. flexuosa*	CGMCC3.19447 T	Roots of *S. officinarum*	China	MN215663	MN264096	MN263957
*C. gladioli*	CBS 210.79	Gladiolus leaf	Romania	HG778987	HG779123	–
*C. geniculata*	CBS 187.50	*Andropogon sorghum* seed	Indonesia	KJ909781	KM083609	KM230410
*C. graminícola*	BRIP 23186 T	*Aristida ingrata*	Australia	JN192376	JN600964	JN601008
*C. guangxiensis*	CGMCC3.19330 T	Roots of *S. officinarum*	China	MN215667	MN264100	MN263961
*C. gudauskasii*	DAOM 165085	Unknown	Unknown	AF071338	AF081393	–
*C. harveyi*	BRIP 57412 T	*Triticum aestivum*	Australia	KJ415546	KJ415400	KJ415446
*C. hawaiiensis*	BRIP 11987 T	*O. sativa*	USA	KJ415547	KJ415399	KJ415445
*C. heteropogonicola*	BRIP 14579 T	*Heteropogon contortus*	India	KJ415548	KJ415398	KJ415444
*C. heteropogonis*	CBS 284.91 T	*H. contortus*	Australia	KJ415549	JN600969	JN601013
*C. hominis*	CBS 136985 T	Human cornea	USA	HG779011	HG779106	–
*C. homomorpha*	CBS 156.60 T	Air	USA	JN192380	JN600970	JN601014
*C. inaequalis*	CBS 102.42 T	Soil	France	KJ922375	KM061787	KM196574
*C. intermedia*	CBS 334.64	*Avena versicolor*	USA	HG778991	HG779155	–
*C. ischaemi*	CBS 630.82 T	*Ischaemum indicum*	Solomon Islands	MH861533	JX276440	–
*C. kenpeggii*	BRIP 14530 T	*Triticum aestivum*	Australia	MH414900	MH433644	MH433662
*C. kusanoi*	CBS 137.29	*Eragrostis major*	Japan	JN192381	LT715862	JN601016
*C. lamingtonensis*	BRIP 12259 T	*Microlaena stipoides*	Australia	MH414901	MH433645	MH433663
*C. lunata*	CBS 730.96 T	Human lung biopsy	USA	JX256429	JX276441	JX266596
*C. malina*	CBS 131274 T	*Zoysia matrella*	USA	JF812154	KP153179	KR493095
*C. manamgodae*	CGMCC3.19446 T	Roots of *S. officinarum*	China	MN215677	MN264110	MN263971
	LC13495	Roots of *S. officinarum*	China	MN215678	MN264111	MN263972
*C. mebaldsii*	BRIP 12900 T	*Cynodon transvaalensis*	Australia	MH414902	MH433646	MH433664
	BRIP 13983	*Cynodon dactylon* x *C. transvaalensis*	Australia	MH414903	MH433647	MH433665
*C. micropus*	CBS 127235 ET	*Paspalum notatum*	Georgia	HE792934	LT715859	–
*C. microspora*	GUCC 6272 T	*Hippeastrum striatum*	China	MF139088	MF139106	MF139115
*C. miyakei*	CBS 197.29 T	*Eragrostis pilosa*	Japan	KJ909770	KM083611	KM196568
*C. mosaddeghii*	IRAN 3131C T	*Syzygium cumini*	Iran	MG846737	MH392155	MH392152
*C. muehlenbeckiae*	CBS 144.63 T	*Sorghum* sp.	USA	MH858242	HG779108	KM196578
*C. neergaardii*	BRIP 12919 T	*O. sativa*	Ghana	KJ415550	KJ415397	KJ415443
	CBS 276.91	Unknown	Australia	LT631362	LT715848	–
*C. neoindica*	IMI 129790 T	*Brassica nigra*	India	MH414910	MH433649	MH433667
*C. nicotiae*	BRIP 11983 T	Soil	Algeria	KJ415551	KJ415396	KJ415442
*C. nodosa*	CPC 28800 T	*Digitaria ciliaris*	Thailand	MF490816	MF490838	MF490859
	CPC 28801	*Brachiaria reptans*	Thailand	MF490817	MF490839	MF490860
*C. nodulosa*	CBS 160.58	*Eleusine indica*	Unknown	JN601033	JN600975	JN601019
*C. oryzae*	CBS 169.53 T	*O. sativa*	Vietnam	KP400650	KP645344	KM196590
*C. ovariicola*	CBS 470.90 T	*Eragrostis interrupta*	Australia	JN192384	JN600976	JN601020
*C. pallescens*	CBS 156.35 T	Air	Indonesia	KJ922380	KM083606	KM196570
*C. palmicola*	MFLUCC 14-0404 T	Dead branches of *Acoelorrhaphe wrightii*	Thailand	MF621582	–	–
*C. pandanicola*	MFLUCC 15-0746 T	Dead leaf of *Pandanus* sp.	Thailand	MH275056	MH412748	MH412763
*C. papendorfii*	CBS 308.67 T	*Acacia karroo*	South Africa	KJ909774	KM083617	KM196594
***C. paraverruculosa***	**FMR 17656 T**	**Soil**	**Mexico**	**LR736641**	**LR736646**	**LR736649**
*C. petersonii*	BRIP 14642 T	*D. aegyptium*	Australia	MH414905	MH433650	MH433668
*C. perotidis*	CBS 350.90 T	*Perotis rara*	Australia	JN192385	KJ415394	KM230407
*C. phaeospara*	CGMCC3.19448 T	Roots of *S. officinarum*	China	MN215686	MN264118	MN263980
*C. pisi*	CBS 190.48 T	*Pisum sativum*	Canada	KY905678	KY905690	KY905697
*C. plantarum*	CGMCC3.19342 T	Roots of *S. officinarum*	China	MN215688	MN264120	MN263982
*C. platzii*	BRIP 27703b T	*Cenchrus clandestinum*	Australia	MH414906	MH433651	MH433669
*C. polytrata*	CGMCC3.19338 T	Roots of *S. officinarum*	China	MN215691	MN264123	MN263984
*C. portulacae*	BRIP 14541 T	*Portulaca oleracea*	USA	KJ415553	KJ415393	KJ415440
*C. prasadii*	CBS 143.64 T	*Jasminum sambac*	India	KJ922373	KM061785	KM230408
*C. protuberans*	CGMCC3.19360 T	Leaves of *S. officinarum*	China	MN215693	MN264125	MN263986
*C. protuberata*	CBS 376.65 T	*Deschampsia flexuosa*	UK	KJ922376	KM083605	KM196576
*C. pseudobrachyspora*	CPC 28808 T	*Eleusine indica*	Thailand	MF490819	MF490841	MF490862
*C. pseudolunata*	UTHSC 09-2092 T	Human nasal sinus	USA	HE861842	HF565459	–
*C. pseudorobusta*	UTHSC 08-3458	Human nasal sinus	USA	HE861838	HF565476	–
*C. radici-foliigena*	CGMCC3.19328 T	Roots of *S. officinarum*	China	MN215695	MN264127	MN263988
	LC11956	Roots of *S. officinarum*	China	MN215698	MN264130	MN263991
*C. radicicola*	CGMCC3.19327 T	Roots of *S. officinarum*	China	MN215699	MN264131	MN263992
	LC11953	Roots of *S. officinarum*	China	MN215700	MN264132	MN263993
*C. ravenelii*	BRIP 13165 T	*Sporobolus fertilis*	Australia	JN192386	JN600978	JN601024
*C. reesii*	BRIP 4358 T	Air	Australia	MH414907	MH433637	MH433670
*C. richardiae*	BRIP 4371 T	*Richardia brasiliensis*	Australia	KJ415555	KJ415391	KJ415438
*C. robusta*	CBS 624.68 T	*Dichanthium annulatum*	USA	KJ909783	KM083613	KM196577
*C. rouhanii*	CBS 144674 T	*Syngonium vellozianum*	Iran	KX139030	MG428694	MG428687
*C. ryleyi*	BRIP 12554 T	*Sporobolus creber*	Australia	KJ415556	KJ415390	KJ415437
*C. saccharicola*	CGMCC3.19344 T	Roots of *S. officinarum*	China	MN215701	MN264133	MN263994
*C. sacchari-officinarum*	CGMCC3.19331 T	Leaves of *S. officinarum*	China	MN215705	MN264137	MN263998
*C. senegalensis*	CBS 149.71	Unknown	Nigeria	HG779001	HG779128	–
*C. shahidchamranensis*	IRAN 3133C T	Crude oil contaminated soil	Iran	MH550084	MH550083	–
*C. soli*	CBS 222.96 T	Soil	Papua New Guinea	KY905679	KY905691	KY905698
*C. sorghina*	BRIP 15900 T	*Sorghum bicolor*	Australia	KJ415558	KJ415388	KJ415435
*C. spicifera*	CBS 198.31	*Capsicum anuum*	Cyprus	HF934916	HG779136	–
	CBS 274.52	Soil	Spain	JN192387	JN600979.	JN601023
*C. sporobolicola*	BRIP 23040b T	*Sporobolus australasicus*	Australia	MH414908	MH433652	MH433671
*C. subpapendorfii*	CBS 656.74 T	Soil	Egypt	KJ909777	KM061791	KM196585
***C. suttoniae***	**FMR 10992 T**	**Human leg wound**	**USA**	HE861828	HF565479	**LR736651**
	**FMR 11690**	**Human sphenoid sinus**	**USA**	HE861826	HF565477	**LR736650**
*C. tamilnaduensis*	SZMC 22226 T	Human corneal scraping	India	MN628311	MN628307	MN628303
	SZMC 26758	Human corneal scraping	India	MN628308	MN628304	MN628300
	SZMC 26759	Human corneal scraping	India	MN628309	MN628305	MN628301
*C. thailandicum*	MFLUCC 15-0747 T	Decaying leaves of *Pandanus* sp.	Thailand	MH275057	MH412749	MH412764
*C. trifolii*	CBS 173.55	*Trifolium repens*	USA	HG779023	HG779124	–
*C. tripogonis*	BRIP 12375 T	*Tripogon loliiformis*	Australia	JN192388	JN600980	JN601025
*C. tropicalis*	BRIP 14834 T	*Coffea arabica*	India	KJ415559	KJ415387	KJ415434
*C. tsudae*	ATCC 44764 T	*Chloris gayana*	Japan	KC424596	KC747745	KC503940
	BRIP 10967	Leaf tip blight of *C. gayana*	Australia	KC424604	KC747754	KC503949
*C. tuberculata*	CBS 146.63 T	*Z. mays*	India	JX256433	JX276445	JX266599
*C. umbiliciformis*	CGMCC3.19346 T	Roots of *S. officinarum*	China	MN215711	MN264142	MN264004
*C. uncinata*	CBS 221.52 T	*O. sativa*	Vietnam	HG779024	HG779134	–
*C. variabilis*	CPC 28815 T	*Chloris barbata*	Thailand	MF490822	MF490844	MF490865
	CPC 28816	*Imperata cylindrica*	Thailand	MF490823	MF490845	MF490866
*C. verruciformis*	CBS 537.75	*Lobibyx* sp. feather	New Zealand	HG779026	HG779133	–
*C. verruculosa*	CBS 149.63	*Elaeis guineensis*	Nigeria	HF934909	HG779110	–
	CBS 150.63	*Punica granatum* leaf	India	KP400652	KP645346	KP735695
	CPC 28792	*C. dactylon*	Thailand	MF490825	MF490847	MF490868
	CPC 28809	*E. indica*	Thailand	MF490824	MF490846	MF490867
***C. vietnamensis***	**FMR 17659 T**	**Unidentified dead leaves**	**Vietnam**	**LR736642**	**LR736644**	**LR736647**
	**FMR 11956**	***Sorghum* seed**	**Indonesia**	**LR736652**	**LR736643**	**LR736648**
*C. warraberensis*	BRIP 14817 T	*D. aegyptium*	Australia	MH414909	MH433653	MH433672
*C. xishuangbannaensis*	KUMCC 17-0185 T	Decaying leaves of *Pandanus amaryllifollus*	China	MH275058	MH412750	MH412765

LOREMIPSUM **^1^**ATCC: American Type Culture Collection, Virginia, USA; BRIP: Queensland Plant Pathology Herbarium, Brisbane, Australia; CBS: Culture collection of the Westerdijk Fungal Biodiversity Institute, Utrecht, the Netherlands; CGMCC: China General Microbiological Culture Collection Center, China; CGMCC: China General Microbiological Culture Collection Center, China; DAOM: Plant Research Institute, Department of Agriculture (Mycology), Ottawa, Canada; FMR: Facultat de Medicina, Universitat Rovira i Virgili, Reus, Spain; GUCC: Department of Plant Pathology, Agriculture College, Guizhou University, P.R. China; IMI: International Mycological Institute, Kew, UK; IRAN: Iranian Fungal Culture Collection, Iranian Research Institute of Plant Protection, Iran; KUMCC: Culture Collection of Kunming Institute of Botany, Kunming, China; LC: Personal culture collection held in the laboratory of Prof. Lei Cai, China; MFLUCC: Mae Fah Luang University Culture Collection, Chiang Ria, Thailand; MUCL: Mycothe`que de l’Universite´ Catholique de Louvain, Louvain-la-Neuve, Belgium; SZMC: Szeged Microbiological Collection at the Department of Microbiology, Faculty if Science and Informatics, University of Szeged, Hungary; UTHSC: Fungus Testing Laboratory, Department of Pathology at the University of Texas Health Science Center, San Antonio, Texas, USA. T and ET indicate ex-type and ex-epitype strain. **^2^** Sequences newly generated in this study and novel species proposed are indicated in bold.

For the ML analysis, the best nucleotide substitution model for the combined analysis of ITS, *gapdh* and *tef*1, determined using the MEGA programme, was Kimura 2-parameters with Gamma distribution (K2+G); the combined analysis of these three phylogenetic markers was tested through Incongruence Length Difference (ILD) implemented in the Winclada programme (Farris et al. 1994). ML bootstrap values (bs) ≥ 70% were considered significant.

For the BI phylogenetic analysis, the best nucleotide substitution model was determined using jModelTest (Posada 2008). For the ITS region, we used Kimura 2-parameter with Invariant sites (K80+I), for *gapdh* General Time Reversible with gamma distribution (GTR+G) and for *tef*1 General Time Reversible with invariant sites (GTR+I). The parameter settings used were two simultaneous runs of 5M generations, four Markov chains, sampled every 1000 generations. The 50% majority-rule consensus tree and posterior probability values were calculated after discarding the first 25% of the samples. A posterior probability (pp) value of ≥ 0.95 was considered significant.

Sequence data generated in the present study were deposited in GenBank (Table 1) and the alignments in TreeBASE (http://treebase.org).

### Phenotypic study

Macroscopic characterisation of the colonies was made on PDA, oatmeal agar (OA; oatmeal 30 g, agar 13 g, distilled water 1 litre) and potato carrot agar (PCA; potato 20 g, carrot 20 g, agar 13 g, distilled water 1 litre), after 7 days at 25 °C in darkness. Colours of the colonies in descriptions were based on Kornerup & Wanscher (1978). Cardinal temperatures for growth were obtained on PDA after 7 days in darkness.

Microscopic features were studied from the specimens mounted in Shear’s solution growing on the same media (Madrid et al. 2014). At least 30 measurements were taken for the calculation of conidial and conidiophores length and width ranges, which are also reported as the mean plus or minus standard deviation in the descriptions. Photomicrographs were taken using a Zeiss Axio-Imager M1 light microscope (Zeiss, Oberkochen, Germany) with a DeltaPix Infinity X digital camera.

Nomenclatural novelties and descriptions were deposited in MycoBank (Crous et al. 2004). Ex-type cultures and holotypes, which were dried cultures, were deposited at the Westerdijk Fungal Biodiversity Institute from Utrecht (CBS, The Netherlands).

## Results

BLASTn results with *gapdh* sequences showed that the isolate FMR 17656 was ≤ 97.6%, similar to *C. verruculosa*CGMCC 28792; FMR 11956 and FMR 17659 showed a similarity of 93.31% and 93.6%, respectively, with *C. spicifera*CBS 198.31; and isolates FMR 10992 and FMR 11690 both exhibited a similarity of 94.7% with the ex-type strain of *C. petersonii* (BRIP 14642). Sequence similarity with this marker between FMR 11956/17659 and FMR 10992/11690 was 97%. These values suggested that the unidentified isolates represented putative new species for the genus, which were then confirmed by multi-locus sequence analysis of ITS, *gapdh* and *tef*1 barcodes. The combined analysis included 128 sequences representing 126 taxa in the genus *Curvularia* and these were rooted with *Bipolaris maydis* (CBS 136.29) and *B. saccharicola* (CBS 155.26) (Suppl. material 1: Fig. S1). The alignment comprised a total of 1928 bp (ITS 432, *gapdh* 573 bp and *tef*1 923 bp), including 546 variable sites (ITS 119 bp, *gapdh* 253 bp and *tef*1 174 bp) and 445 phylogenetically informative (ITS 83 bp, *gapdh* 233 bp and *tef*1 129 bp). The unidentified isolates were allocated to three single lineages in the same clade (74/0.99) together with sequences of the ex-type strains of *C. americana* (UTHSC 08-3414), *C. petersonii* (BRIP 14642) and *C. verruculosa* (CBS 150.63), but with enough distance to be considered distinct species. The two clinical isolates (FMR 10992 and FMR 11690) formed a fully-supported clade closely related to isolates FMR 11956 and FMR 17659, which were collected in Indonesia and Vietnam, respectively and to *C. petersonii*. The fifth isolate (FMR 17656) was related to *C. verruculosa* and *C. americana*, but formed an independent and distant branch from the previously-mentioned species.

**Figure 1.**
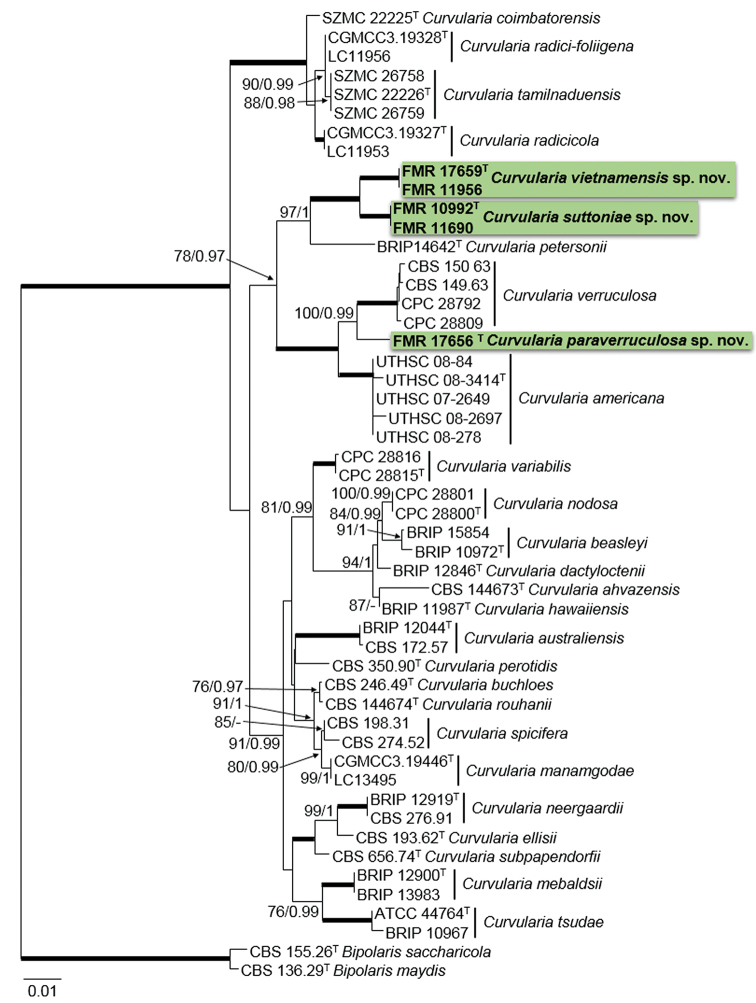
Phylogenetic tree of the *Curvularia* species most related to the new taxa based on Maximum Likelihood analysis obtained by RAxML, using the combined analysis of ITS, *gapdh* and *tef*1 and rooted with *Bipolaris maydis*CBS 136.29 and *Bipolaris saccharicola*CBS 155.26. Bootstrap values (bs) greater than 70% and Bayesian posterior probabilities (pp) greater than 0.95 are given at the nodes (bs/pp). Bold branches indicate bs/pp of 100/1. The novel species are highlighted in bold. Ex-type isolates are marked with a superscript T.

In order to evaluate possible intra- and inter-specific variability within the species and to confirm the novelty of these fungi, we performed a multi-locus analysis, including only those sequences of the species that were more related to the unidentified *Curvularia* isolates (Fig. 1). The alignment comprised a total of 1894 bp (ITS 409, *gapdh* 562 bp and *tef*1 923 bp), with 298 variable sites (ITS 66 bp, *gapdh* 135 bp and *tef*1 97 bp) and 240 being phylogenetically informative (ITS 51 bp, *gapdh* 117 bp and *tef*1 72 bp). The phylogenetic analyses show that these isolates indeed represent three new species, which are described and illustrated in the Taxonomy section. The species can be morphologically differentiated mainly by features of their conidia (Table 2).

**Table 2.** Conidial features of the novel *Curvularia* species proposed here and of their closest relatives.

Species	Size (µm)	Septum no.	Ornamentation	References
*C. americana*	13–28 × 7–15	3–4	Smooth upper cells, verruculose basal cell	Madrid et al. (2014)
*C. palmicola*	23.9–34.7 × 9.3–15.7	3	Smooth	Hyde et al. (2017)
*C. paraverruculosa*	11–37 × 8–12	3(–4)	Verruculose to verrucose	Present study
*C. petersonii*	(15–)17–19(–21) × (5–)5.5–6(–7)	3	Smooth	Tan et al. (2018)
*C. suttoniae*	8–22 × 5–9	(2–)3	Smooth upper cells, verruculose basal cell	Present study
*C. verruculosa*	20–40 × 12–17	3	Rough to verruculose	Sivanesan (1987)
*C. vietnamensis*	15–28 × 5–12	(1–)3(–4)	Smooth	Present study

### Taxonomy

#### Curvularia paraverruculosa

Taxon classificationFungiPleosporalesPleosporaceae

Iturrieta-González, Gené & Dania García sp. nov.

D8C1164A-9DEC-5A3C-9B35-6E3570828FD2

833024

Fig. 2

##### Etymology.

Name refers to the phylogenetic closeness to *Curvularia verruculosa*.

##### Type.

Mexico, Michoacán, Villa Jiménez, from soil, Sept 2016, *E. Rosas de Paz.* (holotype CBS H-24293, culture ex-type FMR 17656, CBS 146220).

##### Description

(PDA at 25 °C). *Mycelium* composed of branched, septate, subhyaline to pale brown, thin- and smooth-walled hyphae, 2–4 μm wide. *Conidiophores* semi- to macronematous, mononematous, septate, straight or flexuous, geniculate at upper part, unbranched or slightly branched, smooth-walled, yellowish-brown to brown, 19–85(–145) × 3–6 μm (av. (±SD) 49.6 (±43.8) × 4.6 (±0.69)). *Conidiogenous cells* terminal or intercalary, polytretic, proliferating sympodially, yellowish-brown, with darkened scars, subcylindrical, 4–6 μm wide. *Conidia* 3(–4)-septate, mostly curved at the third cell from base which is usually larger than the others, sometimes apically bifurcate, verruculose to verrucose, apical and basal cells subhyaline to pale brown, middle cells brown, 11–37 × 8–12 μm (av. (±SD) 24 (±18.38) × 9.58 (±1.66)); hila slightly protuberant, thickened and darkened. Sexual morph not observed.

##### Culture characteristics

(7 d at 25 °C). *Colonies* on PDA reaching 45 mm diam., dark green (30F8), final edge whitish, velvety, flat, margin regular and fimbriate; reverse dark green (30F8). On PCA and OA, reaching 58–60 mm diam., dark green (30F8), final edge whitish, slightly floccose, flat, margin regular and fimbriate; reverse dark green (30F8). Sporulation was abundant on the three media.

##### Cardinal temperature for growth.

Optimum 30 °C, maximum 37 °C, minimum 15 °C.

##### Distribution.

Mexico.

##### Notes.

**Figure 2.**
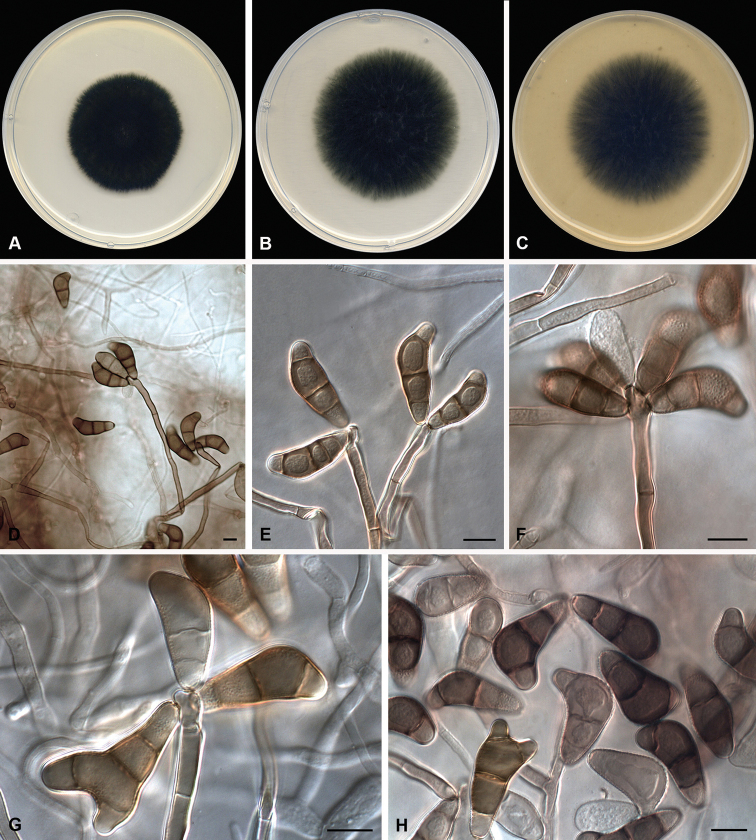
*Curvularia paraverruculosa* sp. nov. (ex-type FMR 17656). **A–C** Colonies on PDA, PCA and OA, respectively, at 25 °C after 7 d **D–H** conidiophores and conidia. Scale bars: 10 μm.

*Curvularia paraverruculosa* is allocated phylogenetically to a strongly-supported clade (100/1) with *C. verruculosa* and *C. americana* (Fig. 1). All three species commonly have 3-septate conidia, but these can be distinguished by their size and ornamentation. Although conidia in *C. verruculosa*, the closest phylogenetic species and *C. paraverruculosa* are entirely verruculose, they are larger in the former (20–40 × 12–17 μm) (Sivanesan 1987). Furthermore, *C. paraverruculosa* also produces apically bifurcate conidia (Fig. 2), which have not been described in *C. verruculosa*. The conidia of *C. americana* are smaller (13–28 × 7–15 μm) and smooth-walled with a slightly verruculose basal cell (Madrid et al. 2014). In addition, microconidiation, described in *C. americana*, has not been observed in *C. paraverruculosa*.

#### Curvularia suttoniae

Taxon classificationFungiPleosporalesPleosporaceae

Iturrieta-González, Wiederhold, Gené & Dania García sp. nov.

D6763586-FEDC-5E6F-9484-1D26368AE9FD

833025

Fig. 3

##### Etymology.

Named in honour of the American mycologist Deanna A. Sutton for her contribution to the body knowledge of microfungi.

##### Type.

USA, Texas, from a human leg wound, 2009, *D.A. Sutton* (holotype CBS H-24294, culture ex-type UTHSC 09-3575, CBS 146221, FMR 10992).

##### Description

(PDA at 25 °C). *Mycelium* consisting of branched, septate, pale brown, smooth-walled to verruculose hyphae, 1–4 µm wide. *Conidiophores* mononematous, semi- to macronematous, erect to slightly flexuous, geniculate at the apex, unbranched or branched, smooth-walled to verruculose, pale brown, 43–103 × 3–5 µm (av. (±SD) 80 (±32.35) × 3.7 (±0.67)). *Conidiogenous cells* terminal, subterminal or intercalary, polytretic, proliferating sympodially, pale brown, darkened scars, subcylindrical to slightly swollen, 3–5 µm wide. *Conidia* (2–)3-septate, straight or curved, with the third cell often larger than the rest, apical and middle cells smooth-walled, basal cell verruculose, pale brown to brown, apical and basal cells paler than the middle cells, 8–22 × 5–9 µm (av. (±SD) 15 (±9.89) × 6.88 (±1.18)); hila protuberant, thickened and darkened. Sexual morph not observed.

**Figure 3.**
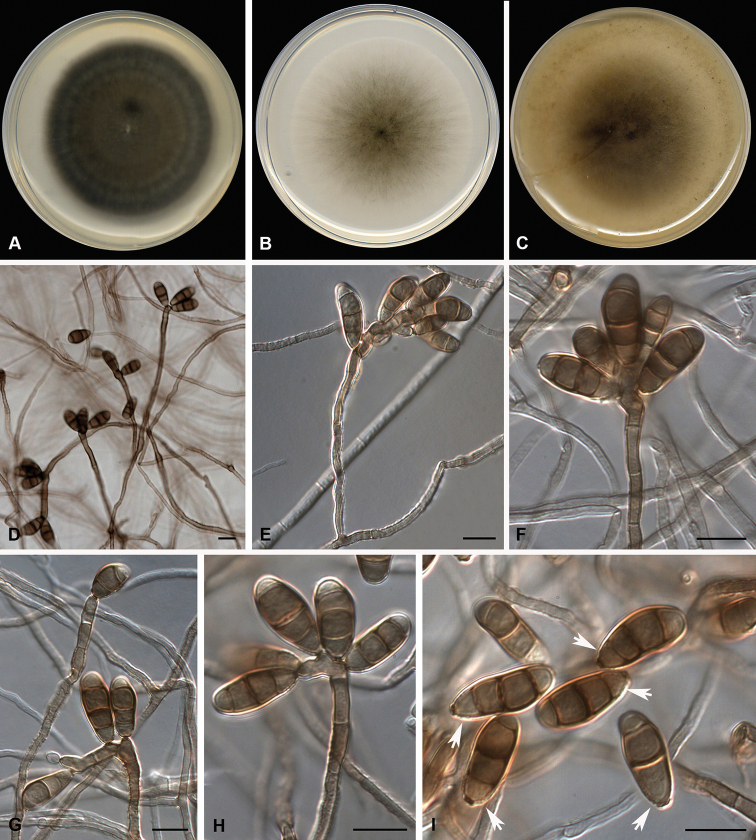
*Curvularia suttoniae* sp. nov. (ex-type FMR 10992). **A–C** Colonies on PDA, PCA and OA, respectively, at 25 °C after 7 d **D–I** conidiophores and conidia with verruculose basal cells (arrows). Scale bars: 10 μm.

##### Culture characteristics

(7 d at 25 °C). Colonies on PDA reaching 66–68 mm diam., yellowish-grey (4B2), velvety, flat, margin slightly irregular and fimbriate; reverse black to brownish-orange (5C4); soluble pigment brown (6E6) present in cultures between 30–37 °C. On PCA, reaching 67 mm diam., olive grey (3D2), slightly floccose at the centre, flat, margin regular and whitish; reverse olive grey (3D2), whitish towards periphery. On OA, reaching 64 mm diam., olive grey (3F2), slightly floccose at the centre, flat, margin regular and whitish; reverse olive grey (3F2). Scarce sporulation on the three media.

##### Cardinal temperature for growth.

Optimum 25–30 °C, maximum 37 °C, minimum 5 °C.

##### Distribution.

USA.

##### Additional specimen examined.

USA, South Carolina, from human sphenoid sinus, 2008, *D.A. Sutton* (UTHSC 08-809, FMR 11690).

##### Notes.

*Curvularia suttoniae* is included in a well-supported clade with *C. petersonii* and *C. vietnamensis*, the latter also described here. Although the three species are clearly differentiated phylogenetically (Fig. 1), they can be distinguished only by subtle morphological features. While the conidia of *C. petersonii* and *C. vietnamensis* are entirely smooth, those of *C. suttoniae* show verruculose basal cells. Furthermore, the conidia in *C. petersonii* are narrower (5–7 µm wide) (Tan et al. 2018) and, in *C. vietnamensis*, they are larger (15–28 × 5–12 μm) than those of *C. suttoniae* (8–22 × 5–9 µm). In addition to these morphological features, *gapdh* sequences easily distinguish the two latter species.

#### Curvularia vietnamensis

Taxon classificationFungiPleosporalesPleosporaceae

Iturrieta-González, Gené & Dania García sp. nov.

1C8CCD40-8C4C-5E77-A738-3C8970080434

833027

Fig. 4

##### Etymology.

Name refers to the country where the species was collected.

##### Type.

Vietnam, north-east region, on an unidentified dead leaf, Aug 2011, *J. Guarro* (holotype CBS H-24295, culture ex-type CBS 146222, FMR 17659).

##### Description

(PDA at 25 °C). *Mycelium* composed of branched, septate, subhyaline to pale brown, thin and smooth-walled to verruculose hyphae, 2–4 μm wide. *Conidiophores* macronematous, mononematous, septate, straight or flexuous, sometimes slightly geniculate at upper part, unbranched to slightly branched, smooth to verruculose, pale brown to brown, 11–136(–194) × 3–6 μm (av. (±SD) 92.2 (±72.86) × 4.21 (±0.85)). *Conidiogenous cells* terminal or intercalary, mono- or polytretic, proliferating sympodially, pale brown, with darkened scars, subcylindrical to swollen, 3–7 μm wide. Conidia (1–)3(–4)-septate, curved, with the third cell from base unequally enlarged, some apically bifurcate, smooth-walled, apical and basal cells pale brown, middle cells brown, 15–28 × 5–12 μm (av. (±SD) 21.38 (± 3.44) × 9.34 (±1.83)); hila slightly protuberant, thickened and darkened. Sexual morph not observed.

**Figure 4.**
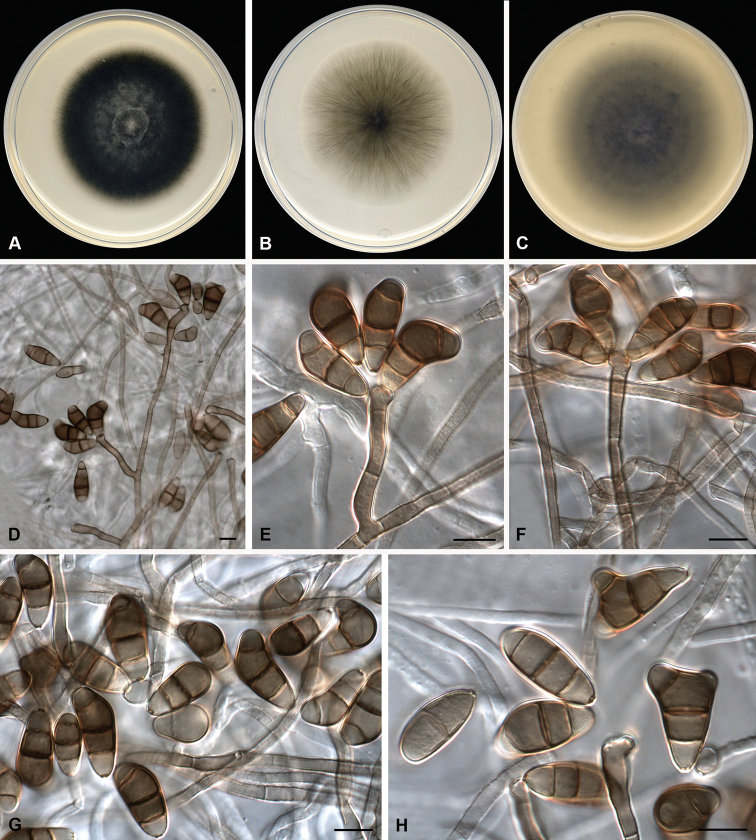
*Curvularia vietnamensis* sp. nov. (ex-type FMR 17659). **A–C** Colonies on PDA, PCA and OA, respectively, at 25 °C after 7 d **D–H** conidiophores and conidia. Scale bars: 10 μm.

##### Culture characteristics

(7 d at 25 °C). *Colonies* on PDA reaching 62 mm diam., greenish-grey to dark green (28C2/29F8), final edge white, umbonate, densely floccose, margin regular; reverse grey (29F1), final edge pale grey (1B2). On PCA, reaching 58 mm diam., olive grey to grey (3F2/3B1), slightly floccose at the centre, margin regular, final edge whitish; reverse olive grey to grey (3F2/3B1). On OA, reaching 74 mm diam., olive (2F3) slightly floccose at the centre, margin regular, flat; reverse olive to greenish-grey (2F3/1C2). Sporulation abundant mainly on PCA and OA.

##### Cardinal temperature for growth.

Optimum 30 °C, maximum 37 °C, minimum 15 °C.

##### Distribution.

Indonesia and Vietnam.

##### Additional specimen examined.

Indonesia, from Sorghum seed, 1948, *J. van der Vecht* (CBS 188.48 = FMR 11956).

##### Notes.

See *C. suttoniae* described above.

## Discussion

As in other Pleosporalean genera, *Curvularia* is currently a well-delineated genus on the basis of molecular data (Manamgoda et al. 2015, Marin-Felix et al. 2017a). However, morphological features and analyses of the ITS barcode are insufficient to accurately identify *Curvularia* species. Thus, the multi-locus sequence analysis of different gene markers (i.e. LSU, ITS, *gapdh*, *rpb*2 and *tef*1) has been used to study the species diversity in *Curvularia* and phylogentic relationships with other similar genera (Hernández-Restrepo et al. 2018, Manamgoda et al. 2012, 2015, Madrid et al. 2014, Marin-Felix et al. 2017a, 2017b, Tan et al. 2018). Marin-Felix et al. (2017a) regarded ITS, *gapdh* and *tef*1 as the DNA barcodes for species delineation in the genus. During the last three years, numerous new *Curvularia* species have been introduced (Hyde et al. 2017, Marin-Felix et al. 2017a, 2017b, Dehdari et al. 2018, Heidari et al. 2018, Liang et al. 2018, Mehrabi-Koushki et al. 2018, Tan et al. 2018, Tibpromma et al. 2018, Kiss et al. 2019, Raza et al. 2019, Zhang et al. 2020). Novel species are found, not only on fresh material collected in various geographical regions, but also in re-evaluation of *Curvularia* isolates deposited in fungal collections and earlier identified by morphological features or ITS sequence analysis.

The five isolates, studied here, showed morphological similarity with *C. americana* or *C. lunata* (Sivanesan 1987, Madrid et al. 2014), but they also showed subtle variations that did not match with these species. Multi-locus analysis of the recommended barcodes facilitated the delineation of the novel species *C. paraverruculosa*, *C. suttoniae* and *C. vietnamensis*, which were closely related to the known species *C. americana*, *C. petersonii* and *C. verruculosa* (Fig. 1).

As in the case of *C. suttoniae*, other related species, such as *C. americana* and *C. verruculosa*, have also been associated with clinical specimens previously (da Cunha et al. 2013, Madrid et al. 2014). However, the role of all these fungi in human diseases has never been proven. Contrary to that, the recently described species *C. coimbatorensis* and *C. tamilnaduensis* were shown to be causal agents of fungal keratitis in India (Kiss et al. 2019). These two latter species, as with *C. suttoniae* and *C. vietnamensis* in our case, could only be molecularly differentiated by *gapdh* and *tef*1 loci; ITS sequence similarity between *C. coimbatorensis* and *C. tamilnaduensis* was 99% (Kiss et al. 2019) and between *C. suttoniae* and *C. vietnamensis*, it was 100%. Therefore, considering clinical laboratories commonly use ITS barcode for fungal diagnosis, not only will the diversity of *Curvularia* species remain obscure in the clinical setting, but also, subsequently, the epidemiology of its species associated with human or animal diseases. Our results suggest that *gapdh* and *tef*1 loci could be good alternatives as barcodes for *Curvularia* identification, since both have a high discriminatory power amongst species. However, *gapdh* would be the recommended locus because there are more sequences available for different species in the genus.

The ITS analysis revealed that *C. palmicola*, only known for its type specimen found on dead branches of *Acoelorrhaphe wrightii* in Thailand (Hyde et al. 2017), is also closely related to the novel species described here. However, this fungus was not included in our concatenate analysis since sequences of *gapdh* and *tef*1 were not available for comparison. Nevertheless, *C. palmicola* can be distinguished morphologically from our species mainly by having conidia with constricted wall at the septum level. Furthermore, *C. palmicola* has longer conidia (23.9–34.7 µm) than *C. suttoniae* (8–22 µm) and C. *vietnamensis* (15–28 µm) and it differs from *C. paraverruculosa* by its smooth-walled conidia.

Despite the fact that DNA sequence analysis is currently mandatory for *Curvularia* identification, two species were recently characterised exclusively, based on morphological data and host association, i.e. *C. tremae* on living leaves of *Trema orientalis* (Haldar 2017) and *C martyniicola* on *Martynia annua* (Kumar and Singh 2018), both from India. *Curvularia tremae* produces up to 4-septate and larger conidia (average length 152.21 μm and 67.75 μm wide at the broadest part) than those described here. Despite the conidia being mostly 3-sepetate, as in our species, *C. martyniicola* differs by having longer conidiophores (95–200 μm) than those of *C. paraverruculosa* (19–85(–145) μm) and *C. suttoniae* (43–103 μm) and by larger conidia (25–45 × 10–15 μm) than those observed in *C. vietnamensis* (15–28 × 5–12 μm).

## Supplementary Material

XML Treatment for Curvularia paraverruculosa

XML Treatment for Curvularia suttoniae

XML Treatment for Curvularia vietnamensis

## Supplementary materials

Supplementary material 1 Figure S1. Phylogenetic tree of the genus *Curvularia* based on Maximum Likelihood analysis obtained by RAxML, using the combined analysis of ITS, *gapdh* and *tef*1 and rooted with *Bipolaris maydis*CBS 136.29 and *Bipolaris saccharicola*CBS 155.26 Data type phylogenetic tree Explanation note Bootstrap values (bs) greater than 70% and Bayesian posterior probabilities (pp) greater than 0.95 are given at the nodes (bs/pp). Bold branches indicate bs/pp of 100/1. The novel species are highlighted in bold. Ex-type and ex-epitype strain are marked with a superscript T and ET, respectively. https://binary.pensoft.net/file/422148This dataset is made available under the Open Database License (http://opendatacommons.org/licenses/odbl/1.0/). The Open Database License (ODbL) is a license agreement intended to allow users to freely share, modify, and use this Dataset while maintaining this same freedom for others, provided that the original source and author(s) are credited.Isabel Iturrieta-González, Josepa Gené, Nathan Wiederhold, Dania García
