# Supplementary figures and images for: Three new Curvularia species from clinical and environmental sources
Source: MycoKeys. 2020 Jun 17;68:1–21. doi: 10.3897/mycokeys.68.51667 (PMC7314867; doi:10.3897/mycokeys.68.51667)

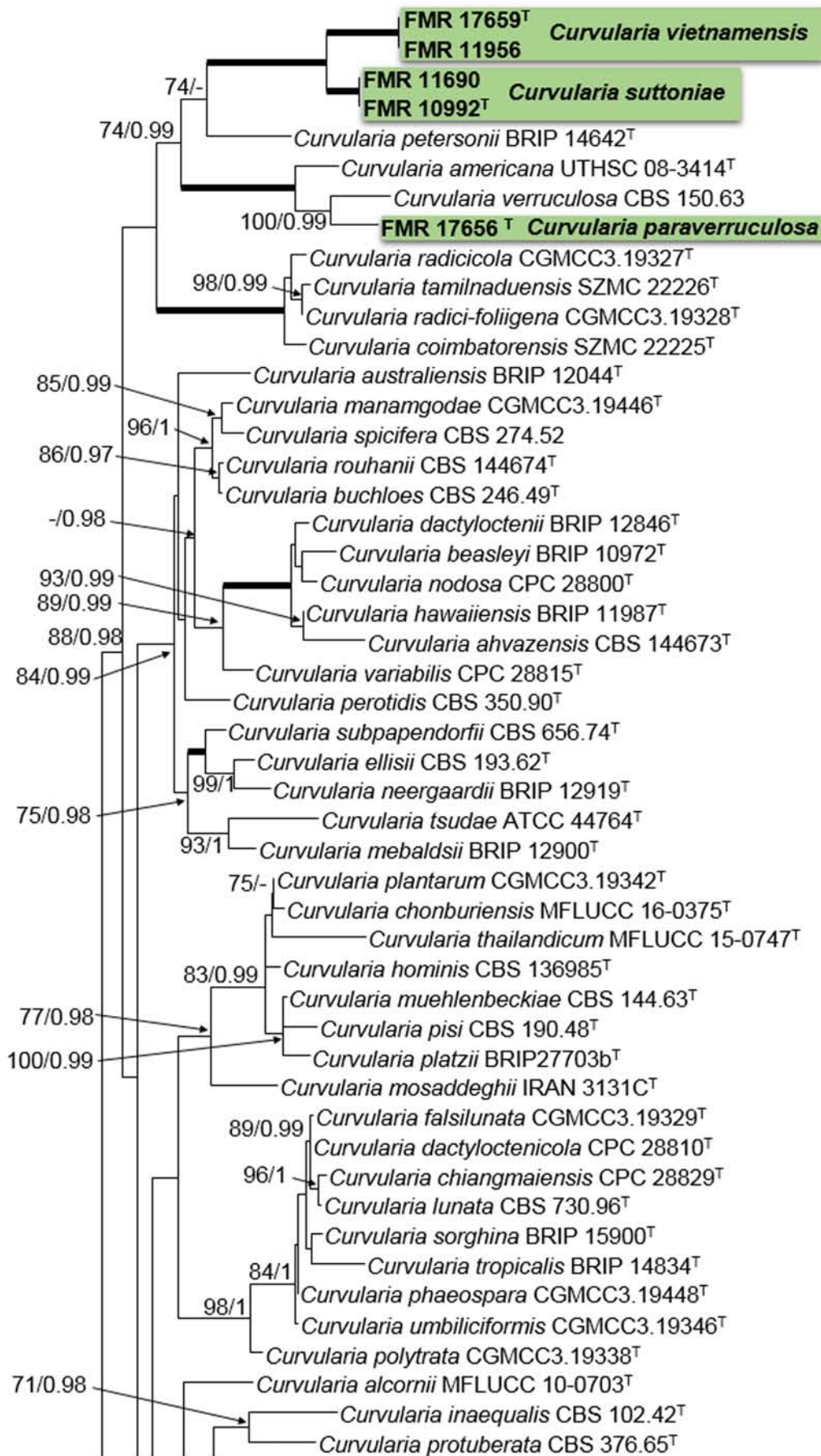

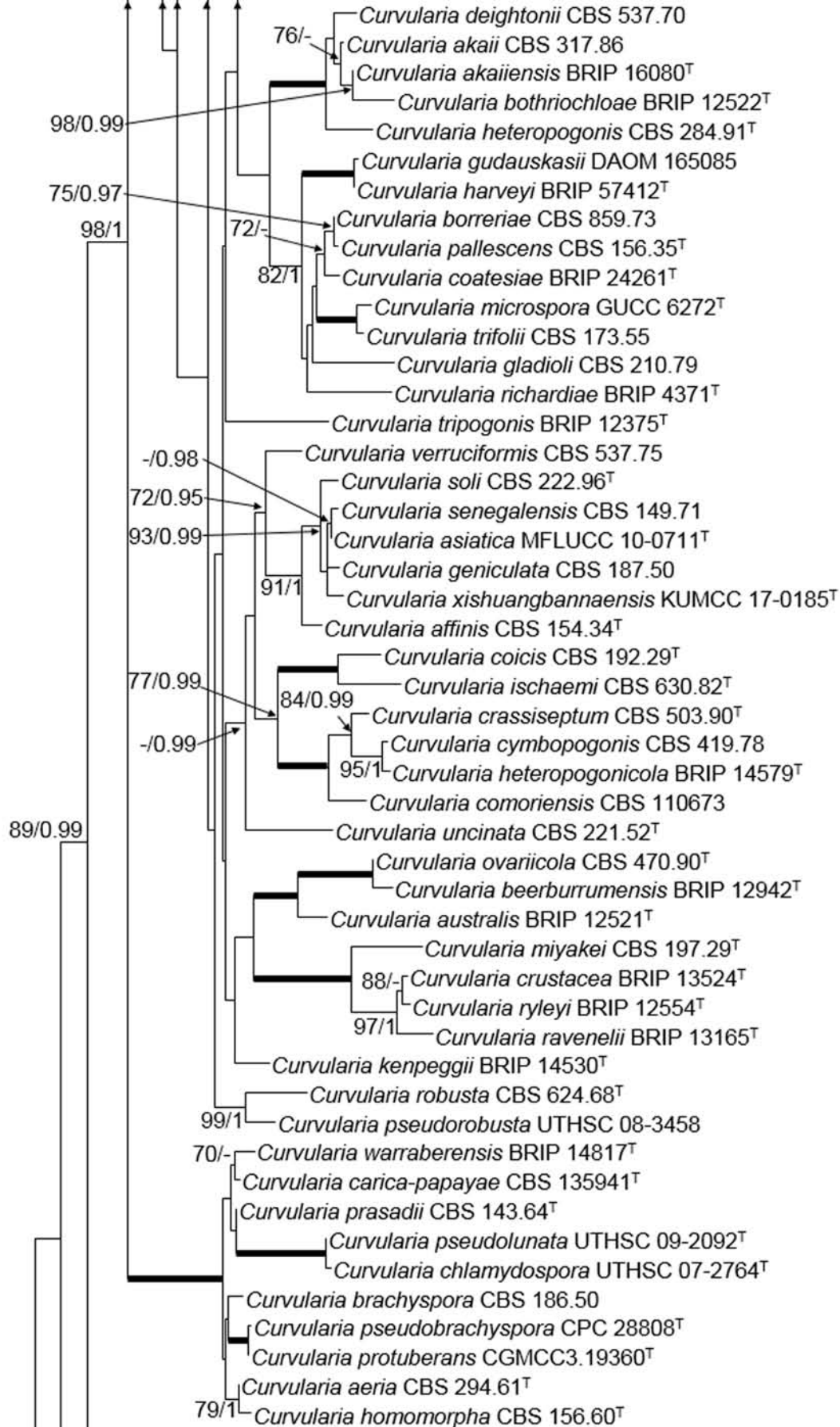

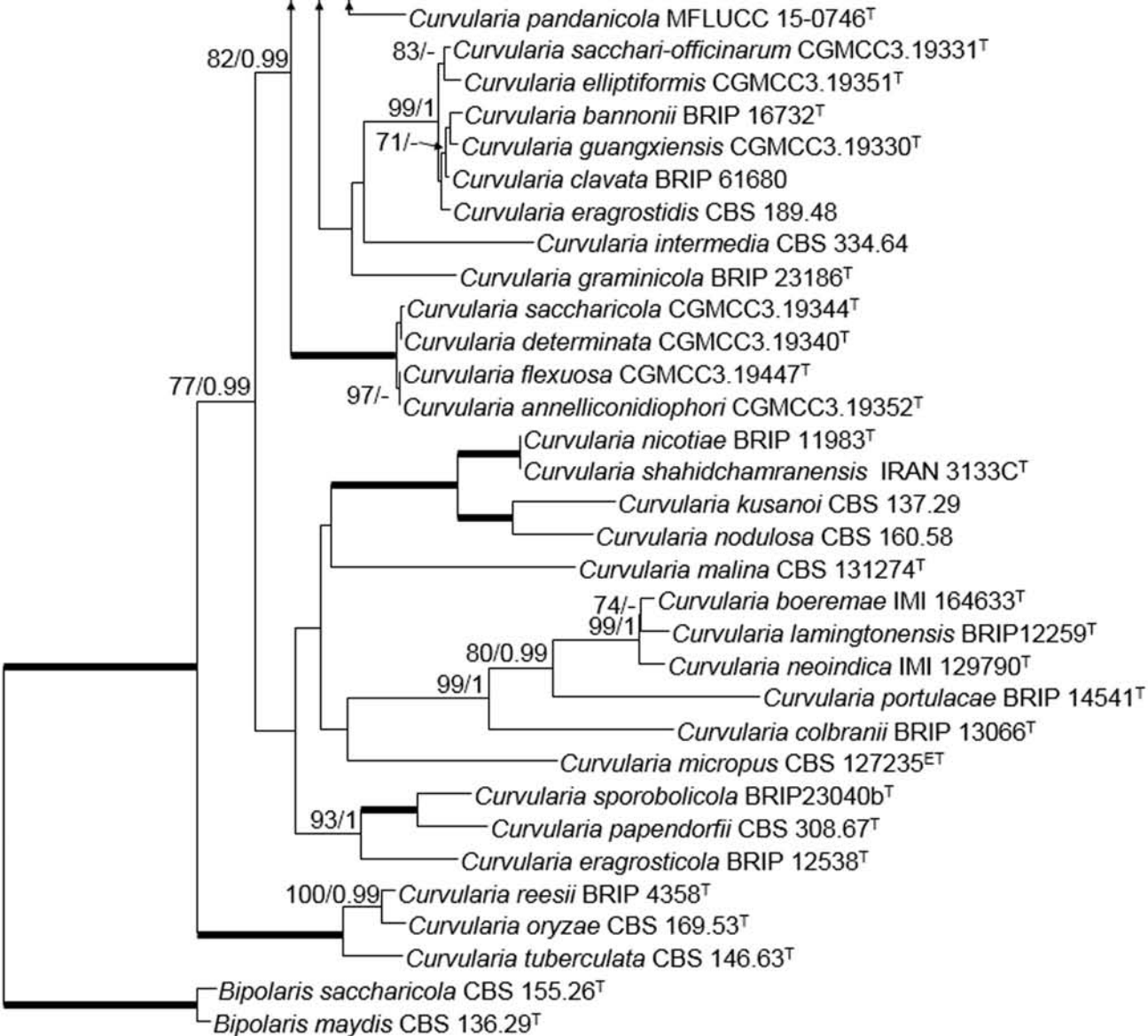

Supplement: Supplementary material 1 — Figure S1. Phylogenetic tree of the genus Curvularia based on Maximum Likelihood analysis obtained by RAxML, using the combined analysis of ITS, gapdh and tef1 and rooted with Bipolaris maydisCBS 136.29 and Bipolaris saccharicolaCBS 155.26 [file mycokeys-68-001-s001.pdf]
